# Anticholinesterases Traits Inbuilt in *Buxaceae* Plant Extracts against Alzheimer’s Disease

**DOI:** 10.2174/1570159X23666250326091016

**Published:** 2025-04-08

**Authors:** Jiri Patocka, Srishti Sharma, Zdenka Navratilova, Namrata Singh, Romana Jelinkova, Nigar Mehboob, Patrik Oleksak, Eugenie Nepovimova, Kamil Kuca

**Affiliations:** 1 Institute of Radiology, Faculty of Health and Social Studies, Toxicology and Civil Protection, University of South Bohemia Ceske Budejovice, Ceske Budejovice, Czech Republic;; 2 Biomedical Research Centre, University Hospital Hradec Kralove, Hradec Kralove, Czech Republic;; 3 School of Studies in Chemistry, Pt. Ravishankar Shukla University, Raipur, (C.G.), 492010, India;; 4 Department of Botany, Faculty of Science, Charles University, Prague, Czech Republic;; 5 Ramrao Adik Institute of Technology, DY Patil University, Nerul, Navi Mumbai, India;; 6 Department of Chemistry, Faculty of Science, University of Hradec Kralove, Hradec Kralove, Czech Republic;; 7 NBC Defence Institute, University of Defence, Vyskov, Czech Republic;; 8 Department of Pharmacy, School of Pharmacy, DY Patil University, Navi Mumbai, Maharashtra, 400706, India;; 9 Center of Advanced Innovation Technologies, VSB-Technical University of Ostrava, Ostrava-Poruba, 708 00, Czech Republic

**Keywords:** Buxaceae, cholinesterase inhibitors, Alzheimer’s disease, inhibition, acetylcholinesterase, butyrylcholinesterase

## Abstract

This review provides a comprehensive account of advances in the field of cholinesterase inhibitors isolated from the Buxaceae family. Naturally occurring anticholinesterases derived from plants are considered to be a potential source of new drug candidates for treating Alzheimer’s disease (AD). AD is now universally accepted as an irreversible, incurable, and progressive neurological disorder. Initiating with memory impairment, propagating with cognitive deficit, and ultimately leading to death is the general pathway of AD. Lower level of acetylcholine in the brain is characterized as one of the prominent reasons for AD. The cholinergic hypothesis states that elevated levels of acetylcholine in the brain can alleviate symptoms of AD. Steroidal and terpenoidal alkaloids isolated from plants of the Buxaceae family have been reviewed here for their anticholinesterase activity. Most of them have shown *in vitro* inhibition of horse serum butyrylcholinesterase (BuChE, EC 3.1.1.7) and electric eel acetylcholinesterase (AChE, EC 3.1.1.8). Although the general consensus has concluded that cholinesterase inhibitors may alleviate AD symptoms but cannot cure the disease, new drugs are still being sought to improve the quality of life of AD patients. Steroidal and terpenoidal anticholinesterase alkaloids can prove to be a promising group of AChE inhibitors.

## 1 INTRODUCTION

Alzheimer's disease (AD) has been recognized as the most devastating form of dementia in the last decades [1]. With the aging population, the risk factor has been increased even more [2]. AD is now largely affecting the socio-economic background of the world [3]. AD has been known to the research world for a century, but the cure and prevention of this neurological disorder are still under dark [4]. The most renowned theory of this memory impairment is a cholinergic hypothesis. The highlight of this theory is the progressive loss of cholinergic synapses occurring in the hippocampus and neocortex portions of the brain, leading to memory loss [5]. Intracellular hyperphosphorylated tau neurofibrillary tangles (NFT) and extracellular amyloid beta (Aβ) aggregates are the two primary pathogenic characteristics of AD. According to the widely accepted amyloid cascade hypothesis, the accumulation of amyloid beta (Aβ) plaques in the brain following dysregulated APP processing is the main factor contributing to the development of AD. Aβ accumulates due to excessive synthesis and insufficient clearance, resulting in neurotoxicity, oxidative stress, synaptic dysfunction, and ultimately cognitive impairment. Two isoforms of Aβ, Aβ40, and Aβ42, with 40 and 42 amino acids, respectively, make up the majority of amyloid deposits [6]. The tau theory suggests that AD could be brought on by an accumulation of hyperphosphorylated tau protein, which then results in the formation of neurofibrillary tangles (NFTs). Studies have consistently demonstrated a strong correlation between the build-up of NFT and cognitive impairment.

Decreased levels of the neurotransmitter acetylcholine appear to be a critical element in the development of this neurodegeneration, making it a key target for therapeutic approaches to treat AD [7]. According to the cholinergic hypothesis, the inhibition of cholinesterases (acetylcholinesterase, AChE, and butyrylcholinesterase, BuChE), an enzyme that catalyzes acetylcholine and butyrylcholine hydrolysis respectively, increases the levels of neurotransmitter in the brain, thus, improving cholinergic functions in AD patients [8].

AD is characterized by cholinergic synaptic dysfunction, which includes both physical synaptic degeneration and functional deficits. Rather than total structural loss, decreased effectiveness is the main factor affecting synaptic transmission early in the disease. Although the exact mechanisms of action of acetylcholinesterase (AChE) inhibitors are not fully known, they are intended to improve synaptic transmission in the remaining functioning synapses by raising acetylcholine (ACh) levels. Their effectiveness in reducing AD's cognitive symptoms, which may be caused by both postsynaptic and presynaptic pathways, is demonstrated by clinical data. For example, presynaptic nicotinic acetylcholine receptors (nAChRs), which control the release of neurotransmitters, can be modulated by extended ACh presence in the synaptic cleft. Though they enhance cognitive function, current AChE inhibitors like donepezil, rivastigmine, and galantamine have drawbacks such as hepatotoxicity and gastrointestinal problems. These difficulties show how safer and more effective inhibitors that target central cholinergic circuits while reducing peripheral adverse effects are needed [9, 10].

The AChE has long been an attractive target for the rational drug design and discovery of mechanism-based inhibitors because of its role in the hydrolysis of the neurotransmitter acetylcholine [9]. The inhibition of this enzyme is considered a promising approach for the treatment of AD and for other possible therapeutic applications in the treatment of various forms of dementia like Parkinson’s disease, ageing, and myasthenia gravis. Additionally, the role of BuChE in the normal aging and diseased brain still needs to be explored [10]. It has been discovered that BuChE is present in significantly higher quantities in Alzheimer’s plaques than in plaques of normal age-related non-demented brains [11]. Thus, this strengthens the fact that both enzymes are crucial for the anti-Alzheimer‘s drugs.

As cholinesterase inhibitors are an important therapeutic strategy in Alzheimer’s disease, efforts are being made in search of new molecules with anti-AChE activity [12, 13]. The fact that naturally occurring compounds from plants are considered to be a potential source of new inhibitors has led to the discovery of an important number of secondary metabolites and plant extracts with the ability to inhibit the enzymes AChE and BuChE. This would ultimately elevate the levels of the neurotransmitter acetylcholine in the brain, thus improving cholinergic functions in patients with Alzheimer’s disease [14, 15].

Taking into account that cholinesterase inhibitors are an important therapeutic strategy for the treatment of AD, many research groups have focused their studies on naturally occurring compounds from plants as potential sources of either new or more effective cholinesterase inhibitors [16]. Several reviews on the newly discovered cholinesterase inhibitors obtained from plants, fungi and marine organisms have also been published over the last years [17-21]. A large number of such inhibitors have been isolated from medicinal plants [22-25].

One such family comprising the anticholinesterase traits is the Buxaceae family. This family comprises six genera with more than 100 species of flowering plants [26]. Along with extensive traditional medicinal properties; they are also found to be effective in memory-related disorders [27, 28]. Many studies have evidenced that steroidal and terpenoidal alkaloids are the major chemical constituents responsible for the biological activities of the plants of this family [29, 30]. These alkaloids have anticholinesterase properties [31] and are also responsible for the toxicity of plants of genus Buxus [32, 33]. This mini-review reports the *in vitro* inhibition of AChE and BuChE by compounds isolated from the plant extracts of the Buxaceae family.

This mini-review presents a comprehensive account of the advances in the field of cholinesterase inhibitors isolated from the plants of the family Buxaceae. The structures of some important phytoconstituents (collected through www.chemspider.com and other chemical databases) are also presented, and the scope for future research is discussed.

## 2 BOTANICAL FEATURES OF THE BUXACEAE FAMILY

*The* Buxaceae family comprises six genera and about 123 species of flowering plants [34]. The family is distributed across temperate, subtropical, and tropical regions worldwide [35, 36]. They are evergreen shrubs or small to medium-sized trees, rarely sub-shrubs or perennial herbs. The leaves are alternate or opposite, decussate, simple, and often leathery. The flowers are unisexual, axillary, or terminate in spikes, racemes, or clusters. The fruit is either a capsule or drupe, and the seeds are black, shiny, and typically accompanied by a caruncle or aril.

According to Angiosperm Phylogeny Group (APG) IV, the Buxaceae family consists of six genera: *Buxus* (including *Notobuxus*, shrubs or trees), *Pachysandra* (woody herbs), *Sarcococca* (shrubs or small trees), *Styloceras* (shrubs or trees), *Didymeles* (trees), and *Haptanthus* (trees). The genus *Didymeles* is sometimes classified as a separate family Didymelaceae, and the genus *Haptanthus* as Haptanthaceae [37, 38]. Despite detailed research, to the best of our knowledge, clinical investigations in this area are still missing [39].

*Buxus sempervirens* (common box), along with its cultivars, are widely grown as ornamental and hedge plants. *Pachysandraprocumbens* and *Pachysandra terminalis* are grown as ground cover. A few wood species of *Buxus* are hard and dense and thus used for carving, engraving and furniture or instrument making. However, first-class timber for joinery is provided by *Styloceras*.

Until now, steroidal and terpenoid alkaloids from Buxaceae family have been discovered and investigated in several species of *Buxus* (*B. sempervire*ns, *B. hyrcana*, *B. papillosa*, *B. macowanii*, *B. microphylla*, *B. natalensis*, *B. balearica*), *Sarcococca* (*S*. *saligna*, *S. coriacea*, *S. hookeriana, S. ruscifolia, S. vagans*) and *Pachysandra* (*P. terminalis, P. procumbens, P. axillaris*).

*Buxus sempervirens* (boxwood, common box, or European box) is an evergreen shrub or small tree native to western, middle, and southern Europe, northern Africa, and Asia Minor. Common box is widely used as ornamental and hedge plants. During the Middle Ages, it was used as a medicinal plant. It was used as a purgative to induce sweating, destroy worms, relieve pain, and treat sexual diseases, skin diseases, and joint inflammations [40]. *Buxus hyrcana* is considered to be a *synonym* of *Buxus sempervirens* subsp. *hyrcana*.

*Buxus papillosa,* locally known as Shamshad, is an evergreen compact shrub distributed in Western Himalaya and traditionally used to cure malaria, rheumatism, skin diseases, and headaches. It is also considered useful as an anti-diarrheal, anti-secretory, cardiotonic, and neurotonic agent [41].

*Buxus macowanii* (Cape box) is a small evergreen tree native to the Eastern Cape Forest of South Africa. This plant is used by local healers to treat wounds and pains. The wood of *B. Macowanii* is used for clay-modeling tools, musical instruments, *etc*. [42, 43].

*Buxus natalensis* (Natal box) is an evergreen shrub or small tree native to South Africa. It is grown as an ornamental plant, the bark is used to enhance the memory of elderly people by traditional healers in the local tribes of South Africa [44].

*Buxus microphylla* (Chinese box) is an evergreen shrub native to southern China. It is used as an ornamental plant, and the leaves are used in folkloric medicine for the treatment of tumors, stomach aches, hernia, and acute myocardial ischemia [45, 46].

*Buxus balearica* (Balearic box) is an evergreen shrub, rarely a small tree. It is native to the western Mediterranean and belongs to endangered species [47].

*Sarcococca saligna* syn. *S. pruniformis* (sweet box or Christmas box) is a small shrub native to northern Pakistan. In Pakistan, it is locally referred to as 'sheha. In traditional medicine of Pakistan, the leaves of *S. saligna* are used as laxative, blood purifier and muscular analgesic [48, 49].

*Sarcococcacoriacea* is an evergreen shrub widely distributed in central Nepal. The extracts and compounds isolated from Sarcococca are used in traditional medicine of Nepal [50, 51].

*Sarcococca hookeriana* (Himalayan sweet box) is an evergreen shrub widely distributed from eastern to western Nepal, northern Assam, southern Tibet, and Bhutan. Rural communities in Nepal have been using the root extracts of this plant against gout. *S. Hookeriana* is also used as an ornamental plant [52].

*Sarcococcaruscifolia* (fragrant sweet box) is a dense evergreen shrub with white fragrant flowers. It is distributed over southern China. As a Chinese folk medicine, the root of *S. Ruscifolia* is widely used for the treatment of stomach pain, rheumatism, bruises, trauma, dizziness, palpitation, and sore throat [53, 54].

*Sarcococcavagans* syn. *S. Balansae* is an evergreen shrub distributed over southern China, Myanmar, and Vietnam. In traditional Chinese medicine, it is used as an anti-tumor agent [55].

*Pachysandraterminalis* (Japanese pachysandra, carpet box, or Japanese spurge) is an evergreen subshrub distributed in China and Japan. Pachysandra is widely used as a groundcover plant. Whole plant has been used as a traditional medicine against pain and stomach ache [56-58].

*Pachysandraprocumbens* (Allegheny pachysandra or Allegheny spurge) is a semi-evergreen subshrub native to southeast parts of North America. It is grown as a groundcover plant [59, 60].

*Pachysandraaxillaris* is an evergreen subshrub distributed in southern parts of China. In folk medicine it is used for treatment of pain and stomach trouble [61].

## 3 ANTICHOLINESTERASE COMPOUNDS OF THE FAMILY BUXACEAE

Not all steroidal and terpenoidal alkaloids isolated from plants of the Buxaceae family have been tested for anticholinesterase activity. If tested, they have, in the vast majority of cases, shown to *in vitro* inhibit horse serum butyrylcholinesterase (BuChE, EC 3.1.1.7) and electric eel acetylcholinesterase (AChE, EC 3.1.1.8). The concentrations of test compounds that inhibited the hydrolysis of substrates (acetylthiocholine and butyrylthiocholine) by 50% (IC_50_) were determined by monitoring the effect of increasing concentrations of these compounds on the inhibition values. The information on the isolation [62] and structure-activity relationship of these cholinesterase inhibitors has also been reported [63].

The results are summarized in six Tables. Table **1**, compounds isolated from *Buxus sempervirens*. Table **2**, compounds from *Buxus papillosa*, Table **3**, compounds from *Buxus macowanii*, Table **4**, compounds from *Buxus natalensis*, Table **5**, compounds from *Sarcococca saligna,* and Table **6**, compounds from *Sarcococca hookeriana* and *Sarcococcacariacea*. Based on the speed at which new anticholinesterase active substances are discovered in the Buxaceae family, it can be judged that their number will increase in the near future [64-78].

### 3.1 *Buxus sempervirens*

Alkaloids from *Buxus sempervirens* are known to have remarkable inhibitory activity above 50% inhibition rate on AChE at 1 mg/ml. Their extracts have individually exhibited higher activity against AChE and BuChE. This positively indicates that these extracts might be interacting with the enzymes in different mechanisms. Anti-AChE and anti-BuChE activities of compounds isolated from *Buxus sempervirens* are shown in Table **1**, along with their structures. Only spirofornabuxine, 17-Oxo-3-benzoylbuxadine, buxhyrcamine, and homomoenjodaramine were observed to exhibit significant AChE inhibition (IC_50_ = 6.3, 17.6, 18.2 and 19.5 µM, respectively). Spirofornabuxine and 17-Oxo-3-benzoylbuxadine are selective inhibitors of AChE (selectivity = 0.05 and 0.09, respectively). On the contrary, 31-demethylcyclobuxoviridine was found to elicit strong and selective BuChE inhibition.

### 3.2 *Buxus papillosa*

*Buxus papillosa* has steroidal alkaloids like buxakarachiamine, buxakashmiramine, and buxahejramine, along with four known bases cycloprotobuxine C, cyclovirobuxeine A, and cyclomicrophyline A which possess AChE as well as BuChE inhibitory activities. Their ethanolic leaf extracts are effective in the treatment of various skin as well as neuronal disorders. Anti-AChE and anti-BuChE activities of compounds isolated from *Buxus papillosa* are shown in Table **2** with their chemical structures. Buxakashmiramine, cyclomicrophylline A, cycloprotobuxine C and cyclovirobuxeine A were observed to exhibit strong AChE inhibition (IC_50_ = 0.74, 2.43, 2.73, and 2.05 µM, respectively). Here, Buxakashmiramine was found to exhibit strong BuChE inhibition.

### 3.3 *Buxus macowanii*

The indigenous people of South Africa have used *Buxus macowanii* for administering mental disorder patients, and they have shown weak to moderate activities in bioassays. Here, the Phytoconstituent, Macowanitriene, and 16α-Hydroxymacowanitriene have shown the highest AChE inhibition (IC_50_ = 10.8 and 11.4 µM, respectively).

### 3.4 *Buxus natalensis*

The IC_50_ value of AChE inhibitory activity of methanolic extract of B. natalensis has been evaluated as 28 µg/ml. Thus, this plant is active in AChE inhibitory assay. Phytochemical investigations of this shrub have also displayed antimicrobial activity. As this shrub is native to South Africa, their traditional healers admit that this enhances the memory of elderly people. Here, O^2^-Natafuranamine, O^10^-Natafura-namine, and Buxafuranamide are found to elicit strong AChE inhibition (IC_50 =_ 3.0, 8.5 and 14.0 µM respectively).

### 3.5 *Sarcococca saligna*

*Sarcococca saligna* is considered a promising member of the Buxaceae family, as its *in vitro* results have suggested that its alkaloids exhibit anti-AChE activities. Steroidal alkaloids have been isolated from the ethanolic extract of the plant, namely, salignenamides A, C, D, E, and F; 2B-hydroxypachysamine-D; axillarines C and F; sarcorine; N-dimethyl Sara codeine; vaganine; 5,6-dehydrosarconidine; 2-hydroxy salignarine; and 2-hydroxysalignamine. The effectiveness of *S. saligna* could help in the management of AD. The alkaloids of *Sarcococca saligna* have shown prominent inhibition of both the enzymes, *i.e*., AChE and BuChE. This positively indicates the possibility of the formation of nervous-system disorders inhibitors. Molecular Dynamics simulation studies have successfully mentioned hydrophobic interactions inside the aromatic gorge, which has been considered the major stabilizing factor in these complexes. However, no alteration has been noticed in the enzyme structure except the reduction in the flexibility at the gorge. Only Vaganine A, Axillaridine A, and Sarsalignenone were observed to exhibit significant AChE inhibition (IC_50_ = 2.32, 5.21, and 5.83 µM, respectively). On the contrary, 5,6-dehydrosarconidine and Isosarcodine were found to elicit strong BuChE inhibition (IC_50_ = 1.89 and 1.89 µM, respectively).

### 3.6 *Sarcococca hookeriana* ‡ and *Sarcococca coriacea* ‡‡

*S. hookeriana*, also known as a hook plant, is another species of the Sarcococca genus that contains six new alkaloids: hookrianamides D, E, F, G, H, and I. The ethanolic extract results have been observed to exhibit effectiveness in the treatment of AD. Here, Sarcovagenine C ‡ and Hookerrianamide F ‡ were observed to exhibit significant AChE inhibition with IC_50_ = 1.5 and 1.6 µM, respectively. Hookerrianamide I ‡ and Sarcovagenine C ‡ were found to elicit strong and selective BuChE inhibition with IC_50_ = 0.3 µM.

## CONCLUSION

This mini-review summarizes the inhibition activities of AChE and BuChE by steroidal and terpenoid alkaloids from the Buxaceae family. Since long back plant extracts have been examined for the bio-medical aspects. Plant extracts are known to possess various beneficial and essential properties that are helpful in the treatment of several kinds of diseases. The continuous efforts have led to the successful findings of the AChE as well as BuChE inhibitor traits in the extracts of the Buxaceae family. AChE and BuChE inhibitors could specifically help in the prevention of AD as these are the target enzymes involved in this neuronal disorder. The steroidal and terpenoidal anticholinesterase alkaloids of the Buxaceae family are an unobtrusive group of natural ingredients of plant origin. These substances offer a new source of potential AD drugs. These compounds can further be modified by enhancing their permeability and solubility which would eventually upgrade their bioavailability.

## Figures and Tables

**Table 1:** *In vitro* inhibition of BuChE and AChE by compounds isolated from *Buxus sempervirens.*

**Phytoconstituent**	**Structures**	**IC_50_** **(µM) BuChE**	**IC_50_** **(µM) AChE**	**Selectivity^a^**	**References**
Arbora-1,9(11)-dien-3-one	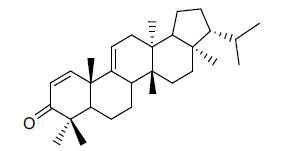	220.1	47.9	0.22	[64]
Buxamine-A and	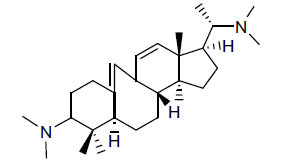	100.2	81.4	0.81	[65]
Buxamine-B	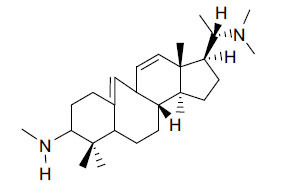	100.5	79.6	0.79	[66]
E-Buxenone	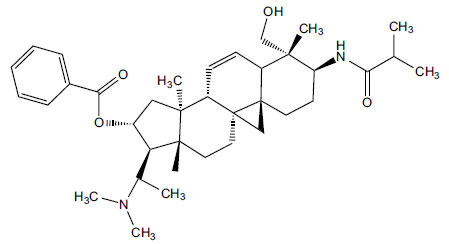	200.7	71.0	0.35	[67]
Z-Buxenone	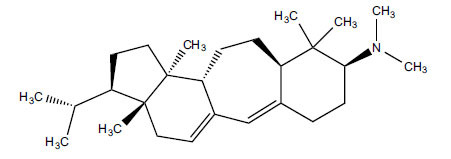	155.8	87.4	0.56	[67]
Buxhyrcamine	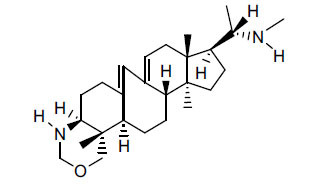	20.9	18.2	0.87	[67]
Buxmicrophylline F	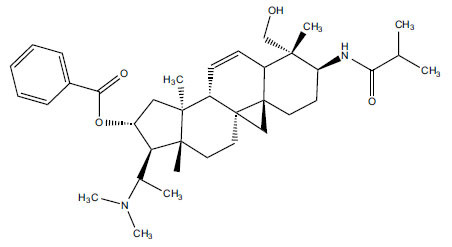	154.2	22.4	0.15	[41]
Buxrugulosamine	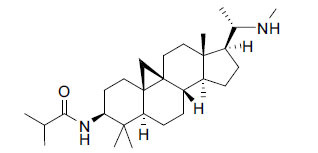	160.2	24.8	0.15	[68]
Cyclobuxophylline O	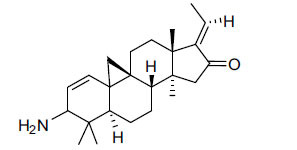	45.0	35.4	0.79	[64]
Cyclobuxoviridine	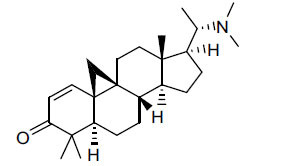	304.5	179.7	0.59	[69]
N_b_-Dimethylcyclobuxoviricine	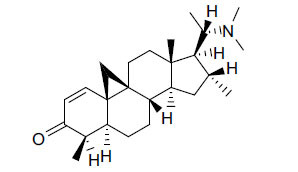	133.8	45.5	0.34	[69]
31-Demethylcyclobuxoviridine	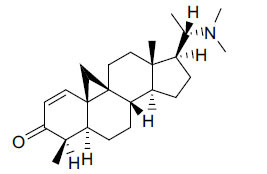	15.4	298.3	19.4	[69]
N^20^-Formylbuxaminol E	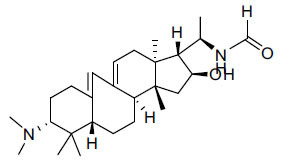	120.9	25.6	0.21	[69, 70]
Homomoenjodaramine	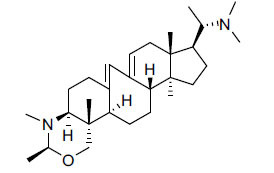	52.2	19.5	0.37	[72]
31-Hydroxybuxamine B	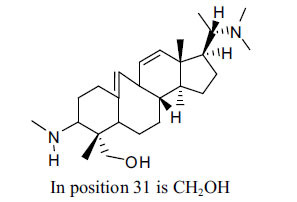	112.1	61.3	0.55	[72]
Moenjodaramine	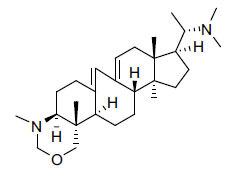	102.4	25	0.24	[71]
17-Oxo-3-benzoylbuxadine	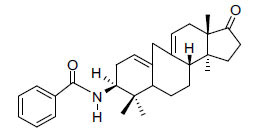	186.8	17.6	0.09	[67]
Papillozine C	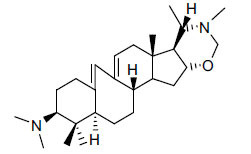	35.2	47.8	1.36	[71]
Spirofornabuxine	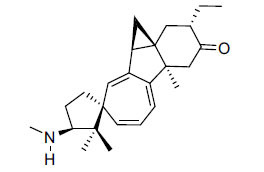	125.2	6.3	0.05	[73]

**Note:** ^a^Selectivity = IC_50 BuChE_/IC_50AChE._

**Table 2:** *In vitro* inhibition of BuChE and AChE by compounds isolated from *Buxus papillosa.*

**Phytoconstituent**	**Structure**	**IC_50_ (µM) BuChE**	**IC_50_ (µM) AChE**	**Selectivity^a^**	**References**
Buxahejramine	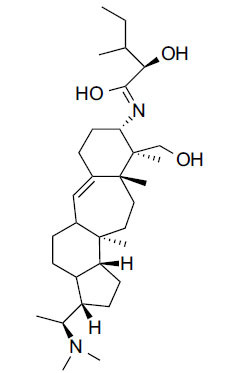	N.D.	162	-	[31]
N,N-Dimethylbuxapapine	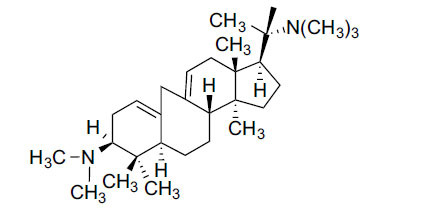	N.D.	7.27	-	[69, 75]
Cyclomicrophylline A	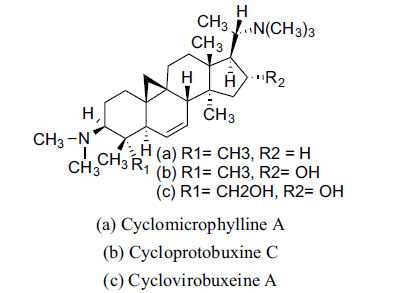	2.43	235	96.7	[31]
Cycloprotobuxine C		2.73	38.8	14.2	[31]
Cyclovirobuxeine A		2.05	105.7	51.6	[31]

**Note:** ^a^Selectivity = IC_50 BuChE_/IC_50 AChE._

**Table 3:** *In vitro* inhibition of BuChE and AChE by compounds isolated from *Buxus macowanii.*

**Phytoconstituent**	**Structure**	**IC_50_ (µM) ** **BuChE**	**IC_50_ (µM) ** **AChE**	**Selectivity^a^**	**References**
Buxbodine	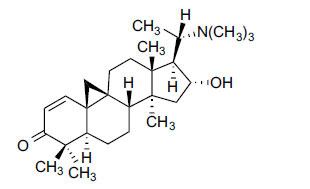	N.D.	50.0	-	[43]
Buxmicrophylline C	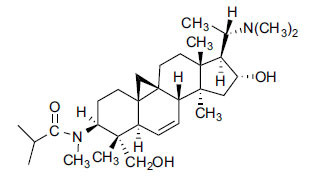	N.D.	20.0	-	[43]
Nb-demethylpapillotrienine	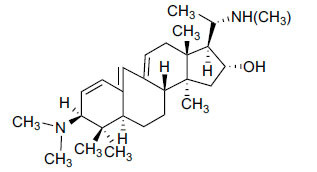	N.D.	19.0	-	[43]
16α-Hydroxymacowanitriene	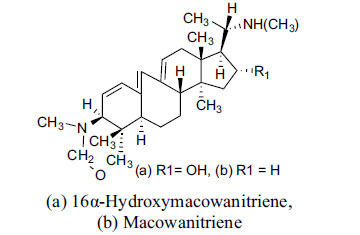	N.D.	11.4	-	[43]
Macowanitriene		N.D.	10.8	-	[43]
Irehine	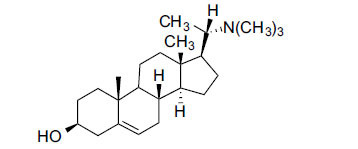	N.D.	98	-	[43]
31-Hydroxybuxatrienone	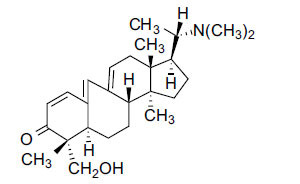	N.D.	17.0	-	[43]
Macowamine	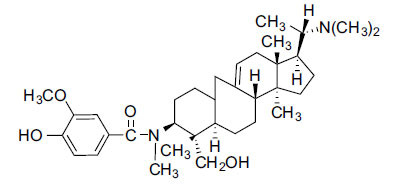	N.D.	45.0	-	[43]
Macowanioxazine	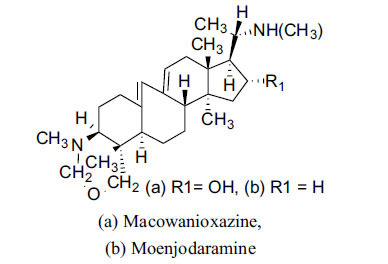	N.D.	32.5	-	[43]
Moenjodaramine		-	27.0	-	[43]

**Note:** ^a^Selectivity = IC_50 BuChE_/IC_50 AchE._

**Table 4:** *In vitro* inhibition of BuChE and AChE by compounds isolated from *Buxus natalensis.*

**Phytoconstituent**	**Structure**	**IC_50_ (µM) BuChE**	**IC_50_ (µM) AChE**	**Selectivity^a^**	**References**
Buxafuranamide	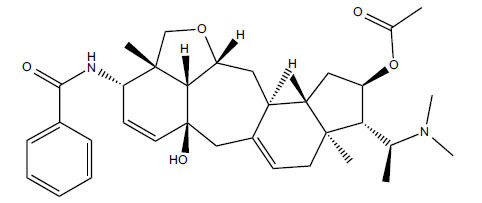	N.D.	14.0	-	[44]
Buxalongifolamide	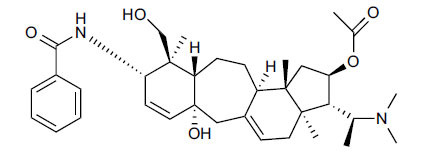	N.D.	30.2	-	[44]
Buxamine A	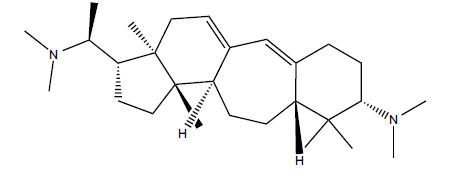	N.D.	80.0	-	[44]
Buxaminol A	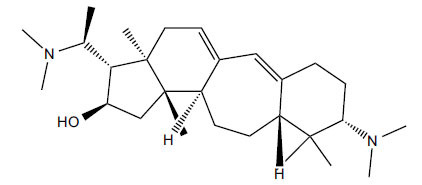	N.D.	29.8	-	[44]
Buxaminol C	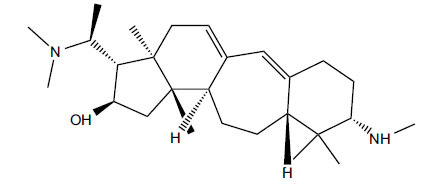	N.D.	40.4	-	[44]
Cyclobuxophylline	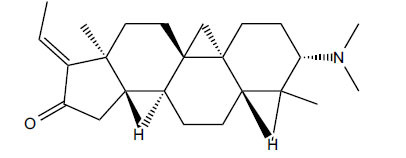	N.D.	58.2	-	[44]
Cyclonataminol	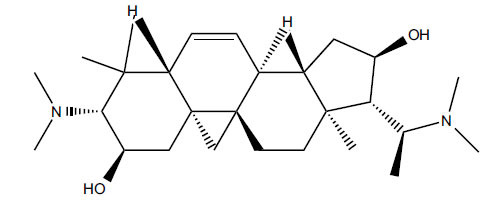	N.D.	22.9	-	[44]
31-Demethylbuxaminol A	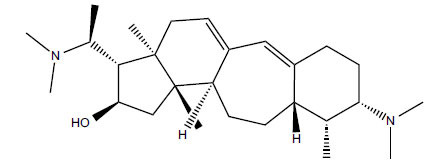	N.D.	25.8	-	[44]
O^2^-Natafuranamine	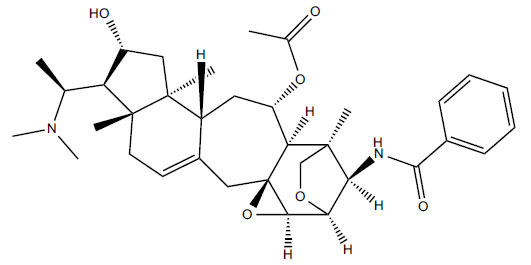	N.D.	3.0	-	[44]
O^10^-Natafuranamine	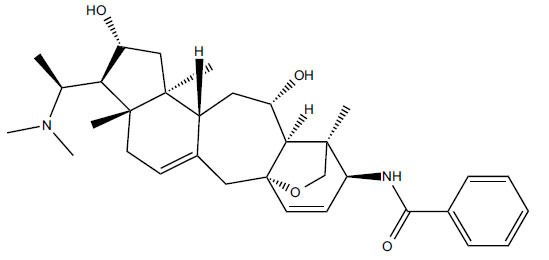	N.D.	8.5	-	[44]

**Note:** ^a^Selectivity = IC_50 BuChE_/IC_50 AChE._

**Table 5:** *In vitro* inhibition of BuChE and AChE by compounds isolated from *Sarcococca saligna.*

**Phytoconstituent**	**Structure**	**IC_50_ (µM) BuChE**	**IC_50_ (µM) ** **AChE**	**Selectivity^a^**	**References**
Alkaloid A	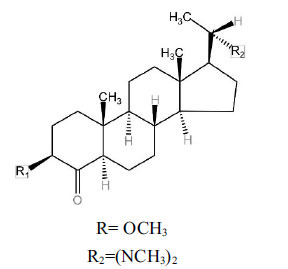	22.13	42.2	1.91	[76]
Axillaridine A	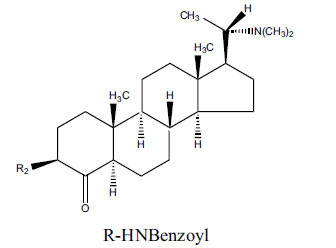	2.49	5.21	2.09	[63]
Axillarine C	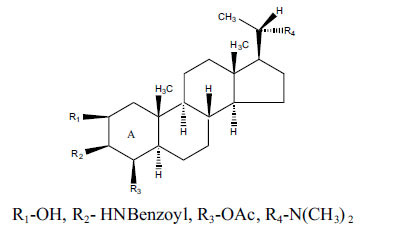	17.99	227.9	12.67	[63]
Axillarine F	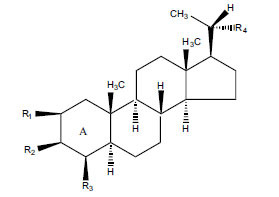	18.24	182.4	10.0	[63]
5,6-dehydrosarconidine	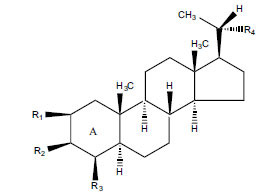	1.89	20.29	10.74	[63]
N_α_-Demethylsaracodine	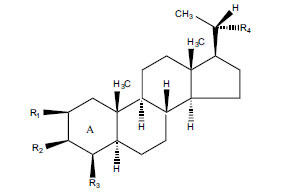	16.55	204	12.33	[63]
Dictyophlebine E	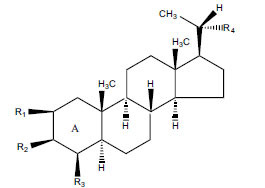	3.65	6.21	1.7	[63]
Epipachysamine D	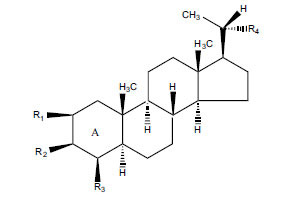	2.82	28.93	10.26	[63]
Iso-N-Formylchoneformine	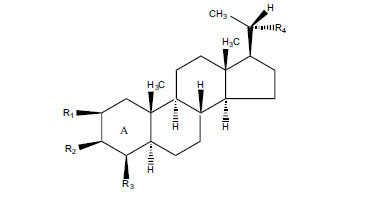	4.07	6.357	1.56	[63]
2b-Hydroxyepipachysamine D	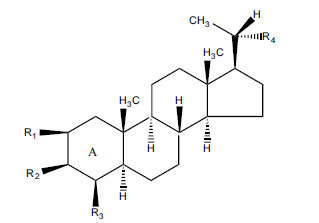	28.96	78.2	2.7	[63]
2-Hydroxysalignamine E	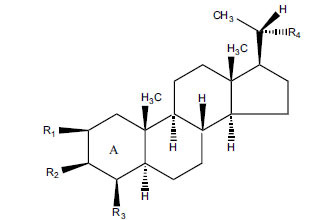	6.91	15.99	2.31	[63]
Isosarcodine	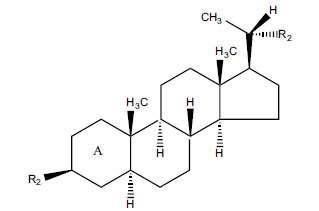	1.89	10.31	5.46	[63]
Saligcinnamide	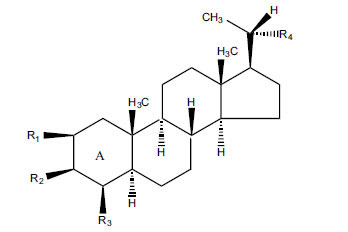	4.84	19.99	4.13	[63]
Salignamine	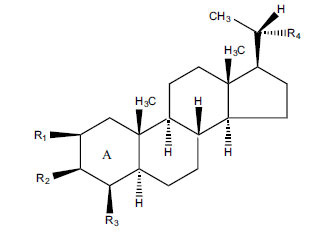	20.95	82.5	4.58	[63]
Salignenamide A	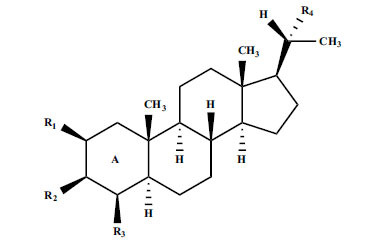	4.63	50.64	4.63	[63]
Salignenamide C	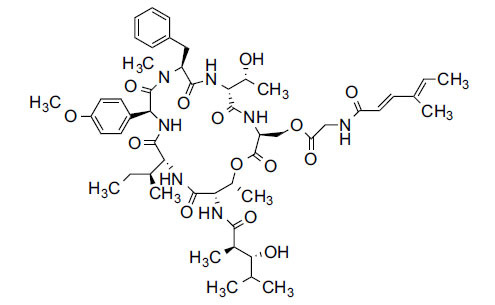	38.36	61.3	1.6	[63]
Salignenamide D	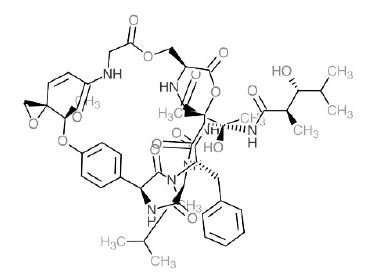	23.78	185.2	7.79	[63]
Salignenamide E	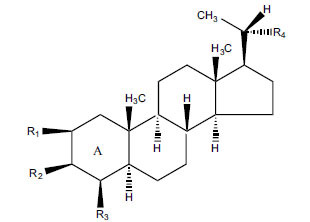	3.65	6.21	1.7	[63]
Salignenamide F	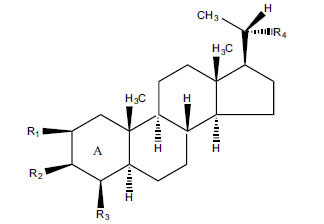	4.07	6.35	1,56	[63]
Salonine A	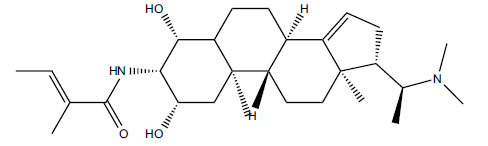	32.7	33.4	1.02	[77]
Salonine B	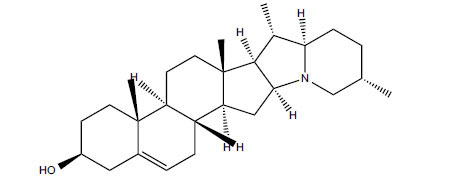	4.5	N.S.	-	[77]
Sarcocine	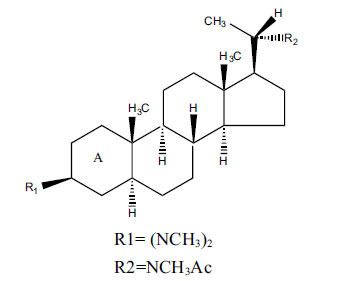	3.86	20.0	5.18	[74]
Sarcodine	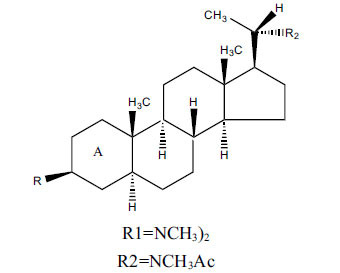	18.31	49.77	2.72	[74]
Sarcorine	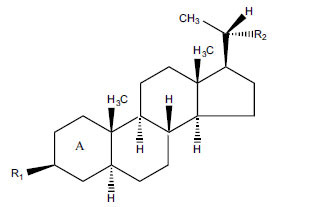	10.33	69.99	6.78	[74]
Sarcodinine	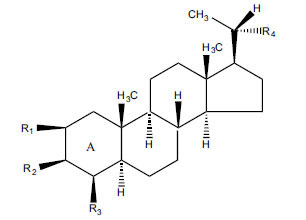	12.51	40.04	3.2	[63]
Sarsalignenone	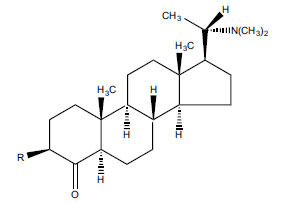	4.29	5.83	1.36	[63]
Sarsalignone	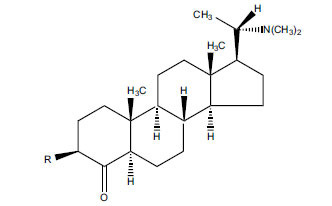	- 2.18	7.02 7.02	- 3.22	[63]
Vaganine A	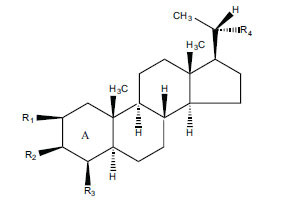	- 8.59	8.59 2.32	- 0.27	[63, 78]

**Note:** ^a^Selectivity = IC_50 BuChE_/IC_50 AChE._

**Table 6:** *In vitro* inhibition of BuChE and AChE by compounds isolated from *Sarcococca hookeriana* ‡ and *Sarcococca coriacea* ‡‡.

**Phytoconstituent**	**Structures**	**IC_50_ (µM) BuChE**	**IC_50_ (µM) AChE**	**Selectivity^a^**	**References**
Dictyophlebine ‡	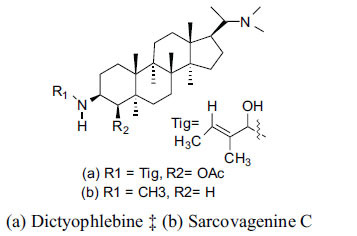	3.6	6.2	1.72	[52]
Sarcovagenine C ‡		0.7 0.3	1.5 8.0	2.14 26.7	[31, 52]
Epoxynepapakistanine A ‡‡	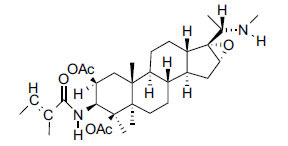	77.4	>200	>2.58	[51]
Funtumarine C ‡‡	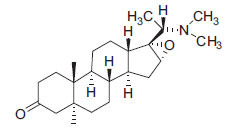	6.56	45.75	6.97	[51]
Hookerrianamide D ‡	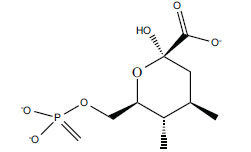	100.2	59.0	58.9	[31]
Hookerrianamide E ‡	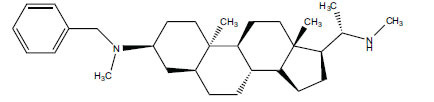	6.0	15.9	2.65	[31]
Hookerrianamide F ‡	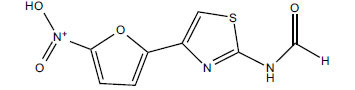	7.2	1.6	0.22	[31]
Hookerrianamide G ‡	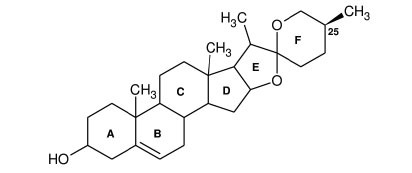	1.5	11.4	7.6	[31]
Hookerrianamide H ‡	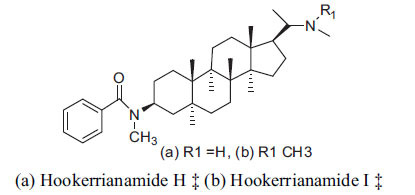	1.9	2.9	1.53	[52]
Hookerrianamide I ‡		0.3	34.1	113.7	[52]
N_α_-Methylepipachysanine D ‡	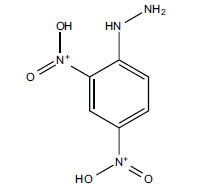	3.2	10.1	3.16	[31]
N-Metylfuntumine ‡‡	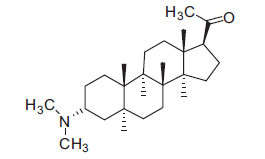	12.69	97.61	7.69	[51]
Saligenamide A ‡	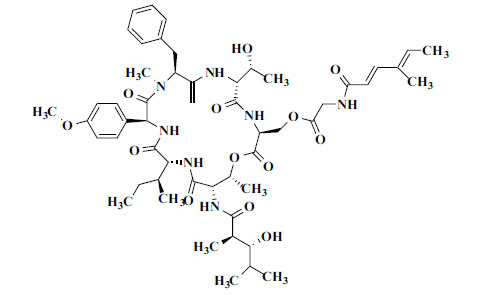	4.6	50.6	11.0	[31]
Sarcovagenine D ‡	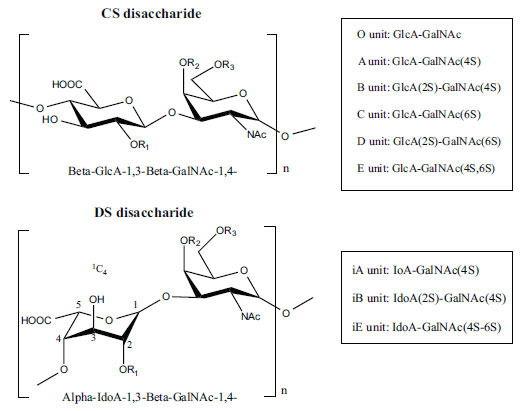	2.3	2.2	0.96	[31]
Terminaline ‡	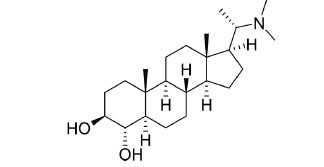	0.6	113.1	188.5	[31]

**Note:** ^a^Selectivity = IC_50 BuChE_/IC_50 AChE._

## LIST OF ABBREVIATIONS

AChE: Acetylcholinesterase
AD: Alzheimer's Disease
Aβ: Amyloid Beta
NFT: Neurofibrillary Tangles
